# An Analytical Model for Estimating Alveolar Wall Elastic Moduli From Lung Tissue Uniaxial Stress-Strain Curves

**DOI:** 10.3389/fphys.2020.00121

**Published:** 2020-02-25

**Authors:** Samer Bou Jawde, Ayuko Takahashi, Jason H. T. Bates, Béla Suki

**Affiliations:** ^1^Biomedical Engineering, Boston University, Boston, MA, United States; ^2^Department of Medicine, Larner College of Medicine, University of Vermont, Burlington, VT, United States

**Keywords:** multiscale, alignment, tensile modulus, bond-bending modulus, aging

## Abstract

The non-linear stress-strain behavior of uniaxially-stretched lung parenchyma is thought to be an emergent phenomenon arising from the ensemble behavior of its microscopic constituents. Such behavior includes the alignment and elongation of randomly oriented alveolar walls with initially flaccid fibers in the direction of strain. To account for the link between microscopic wall behavior and the macroscopic stress-strain curve, we developed an analytical model that represents both alignment and elongation of alveolar walls during uniaxial stretching. The model includes the kinetics and mechanical behavior of randomly oriented elastic alveolar walls that have a bending stiffness at their intersections. The alignment and stretch of the walls following incremental stretch of the tissue were determined based on energy minimization, and the total stress was obtained by differentiating the total energy density with respect to strain. The stress-strain curves predicted by the model were comparable to curves generated by a previously published numerical alveolar network model. The model was also fit to experimentally measured stress-strain curves in parenchymal strips obtained from mice with decreased lung collagen content, and from young and aged mice. This yielded estimates for the elastic modulus of an alveolar wall, which increased with age from 4.4 to 5.9 kPa (*p* = 0.043), and for the elastic modulus of fibers within the wall, which increased with age from 311 to 620 kPa (*p* = 0.001). This demonstrates the possibility of estimating alveolar wall mechanical properties in biological soft tissue from its macroscopic behavior given appropriate assumptions about tissue structure.

## Introduction

The macroscopic mechanical behavior of a biological tissue such as the lung parenchyma arises from the ensemble behavior of its microscopic constituents (Suki and Bates, 2011). This macroscopic behavior is usually very different from the behaviors of the constituents themselves. Furthermore, while macroscopic behavior determines physiological phenotype, disease processes and pharmacotherapies invariably act at the microscale level. It is thus crucial to understand macroscopic mechanical behavior of tissue as an emergent consequence of structure and function at the microscale. In particular, the strain-stiffening behavior of biological soft tissues has been primarily attributed to the way in which load-bearing fibers are recruited via alignment and/or straightening within the extracellular matrix (ECM) (Wuyts et al., 1995; Suki et al., 2005; Suki and Bates, 2011). The progressive recruitment of collagen fibers with increasing strain has been modeled specifically for lung parenchymal tissue (Maksym and Bates, 1997), human vein, fascia, tympanic membrane, rabbit papillary muscle (Decraemer et al., 1980), and ligament (Liao and Belkoff, 1999). Both recruitment and fiber reorientation were simultaneously taken into account by Lanir (1979). Nevertheless, the stress-strain behavior of the individual tissue constituents themselves must also be taken into account for a complete understanding of tissue mechanics. Determining this behavior experimentally is challenging because of the tiny length scales involved, but Sacks was able to determine fiber orientation using small-angle light scattering and numerically solved the constitutive equations to estimate the stress-strain behavior of individual collagen fibers (Sacks, 2003). Furthermore, these fibers do not act in isolation. They interact with each other and with other components of the ECM through electrostatic forces arising, for example, from negatively charged proteoglycans (PGs) that exert repulsive forces on each other (Cavalcante et al., 2005; Ritter et al., 2009).

These models, however, all fail to capture the foam-like structure of lung parenchyma comprised of alveoli and alveolar ducts. Models based on 3-dimensional alveolar geometry have been proposed (Denny and Schroter, 2006; Parameswaran et al., 2011; Pidaparti et al., 2013), but these all assume constitutive properties for the alveolar wall and predict tissue behavior on this basis. Here we seek to do the reverse, namely to infer alveolar wall properties from the macroscopic mechanical behavior of lung parenchyma. Most models of lung tissue that have been proposed to date are limited in this regard because they are numerical (Maksym et al., 1998; Cavalcante et al., 2005; Takahashi et al., 2014), and are thus computationally expensive. Also, the insights provided by numerical models are limited to the particular parameter sets used to calculate examples of model behavior. Analytical models, in contrast, have the potential to provide general insight that is not bound by particular exemplars. Analytical models can also usually be solved much more quickly than numerical models, which makes them more suited to the present inverse modeling application, although they invariably require significant simplifying assumptions in order to be tractable.

Accordingly, the goal of this study was to develop an analytical multi-scale model that links the elastic stiffness and the alignment mechanism of the alveolar walls to the macroscopic mechanical stress-strain behavior of the lung tissue itself. We test the predictions of this model by comparing its macroscopic stress-strain curves to those made by a previously developed numerical model. We also tested model predictions against experimental data obtained in parenchymal tissue strips under uniaxial stretching. These conditions modulate fiber stretching and alignment without being confounded by other phenomena operative in the 3-dimensional expansion of lungs *in situ* such as volumetric expansion, shear, and surface tension (Wilson and Bachofen, 1982; Bachofen and Schürch, 2001; Denny and Schroter, 2006). This enabled us to focus exclusively on the mechanical properties of the tissue fibers and how they change with age, the latter being known to principally affect the fibers (Kohn et al., 2015; Panwar et al., 2015).

## Methods

### Model Development

**Figure 1:**
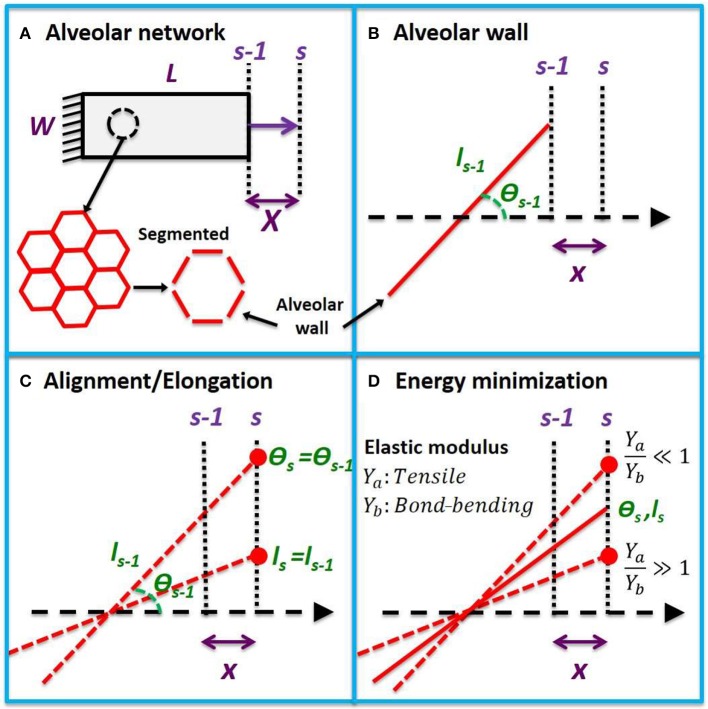
Uniaxial model development. **(A)** Uniaxial stretching of a tissue strip from step *s* − 1 to *s* with an incremental macroscopic displacement *X*. The tissue is composed of a hexagonal network which can be partitioned into alveolar wall segments. **(B)** A single wall segment with length *l*_*s* − 1_ and angle θ_*s* − 1_ with respect to the direction of stretch at step *s* − 1. A microscopic displacement *x* will cause the wall to align by rotation and stretch. **(C)** The alveolar wall will follow the microscopic displacement between two extremes of pure elongation and pure rotation. **(D)** When the tensile modulus of the wall is much larger than its bond-bending modulus (*Y*_*a*_ > *Y*_*b*_), it will rotate, and when its bond-bending modulus is much larger (*Y*_*b*_ >> *Y*_*a*_), it will elongate in order to minimize energy. The new angle (θ_*s*_) and length (*l*_*s*_) at step *s* will lie between these two extremes at a location that minimizes the total elastic energy.

We assume that a 2-dimensional strip of lung tissue with length *L* and width *W* is held in place at one end and stretched uniaxially from the other in a sequence of equal increments. The tissue strip is modeled as a network of hexagonal alveoli. Each of the *N* alveolar walls in the strip (Figure 1A) has a tensile Young's modulus *Y*_*a*_, while adjacent walls are bonded together at intersections that have bond-bending modulus *Y*_*b*_ that resists changes in angle between neighboring walls. The volume fraction of the tissue comprised of alveolar walls is δ_*V,w*_, so the effective tensile and bond-bending moduli of the tissue are *Y*_*ae*_ = *Y*_*a*_ δ_*V,w*_ and *Y*_*be*_ = *Y*_*b*_ δ_*V,w*_, respectively (assuming that whatever fills the alveolar lumen makes no contribution to either). At incremental stretch step *s* − 1, the *n*^th^ alveolar wall has length *l*_*n,s* − 1_ and strain ε_*n,s* − 1_ and is oriented at an angle θ_*n,s* − 1_ with respect to the direction of tissue stretch (Figure 1B).

An incremental displacement *x* of the right-hand end of the alveolar wall in the direction of tissue stretch will, in general, cause the wall itself to undergo some combination of alignment and elongation (Figure 1C). Elongation of the wall increases its elastic energy, while alignment alters the elastic energy stored within its wall-wall bonds (Figure 1D). The configuration of the various walls within the tissue at any time step *s* will be that which minimizes the sum of all these energies. The stress within the tissue is then the derivative of the energy density of the system with respect to macroscopic strain. To establish the global stress-strain equation for the tissue, we further assume

- constant tensile and wall-bending moduli,
- affine tissue deformation characterized by the microscopic incremental tissue strain (*dϵ*_*x*_) due to the microscopic incremental displacement *x* being equal to the macroscopic incremental strain (*dϵ*_*x*_) due to the macroscopic incremental displacement *X* (*dϵ*_*x*_ = *dϵ*_*X*_),
- extension and alignment of the alveolar wall bear a linear relationship to each other when strained incrementally,
- boundary effects are minimal (i.e., *L* ≫ *l*_*s* − 1_ and *W* ≫ *l*_*s* − 1_).

These assumptions lead to the following equation relating macroscopic tissue stress σ_*T*_ at the current stretch step *s* to the alveolar wall constitutive properties, the current strains, angles, and initial angles θ_*n,o*_ for all walls (*n* = 1, …, *N*), and the incremental tissue strain (see Appendix A for a detailed derivation):

(1) $$\begin{array}{l} \sigma_{T}\left(s\right)=Y_{ae}\frac{1}{N}\overset{N}{\underset{n=1}{\sum }}\frac{d\epsilon_{X}+\;\varepsilon_{n,s-1}+\left(1+\varepsilon_{n,s-1}\right)\left(\theta_{n,0}-\theta_{n,s-1}\right)\tan\left(\theta_{n,s-1}\right)}{1+\frac{Y_{ae}}{Y_{be}}{\left(1+\varepsilon_{n,s-1}\right)}^{2}\tan^{2}\left(\theta_{n,s-1}\right)} \end{array}$$

In the limit of large macroscopic stretch, all the alveolar walls become aligned, the denominator of the above equation tends to 1, and the last term of the numerator tends to 0. At this point, the equation reduces to the description of an arbitrary number of linearly elastic walls in parallel thus:

(2) $$\begin{array}{l} \sigma_{T}=Y_{ae}\left(d\epsilon_{X}+\;{\bar{\varepsilon }}_{s-1}\;\right) \end{array}$$

where ${\bar{\varepsilon }}_{s-1}$ is the mean wall strain. The slope of this stress-strain relationship is independent of angle because, at full alignment, the bond-bending energy remains constant. The asymptotically linear portion of the stress-strain curve at high strain thus depends only on *Y*_*ae*_. In general, however, the macroscopic stress-strain relationship expressed by Equation 1 involves the two elastic moduli *Y*_*ae*_ and *Y*_*be*_, which can be estimated by fitting Equation 1 to experimental measurements of σ_*T*_ and ϵ_*X*_. Finally, if the average fiber volume fraction (δ_*V,f*_) within the alveolar wall is known, and assuming that the fibers are the major contributors to the wall tensile modulus, then the average elastic modulus of a single fiber (*Y*_*f*_) can be estimated as.

(3) $$\begin{array}{l} Y_{f}≅\frac{Y_{a}}{\delta_{V,f}}≅\frac{Y_{ae}}{\delta_{V,w}\delta_{V,f}} \end{array}$$

#### Computational Model Validation

We compared the predictions of the above model to stress-strain relationships generated by a previously described numerical model. The numerical model, introduced by Cavalcante et al. (2005), consists of a hexagonal cell network representing a strip of lung parenchyma, where each hexagonal cell represents an alveolus in cross-section and each hexagonal segment represents an alveolar wall. (Figure 9A in Appendix B). Each wall is modeled as a spring with spring constant *k* in units of [N/m]. The relative rotation of two alveolar walls about their point of intersection is hindered by a bond-bending spring with an elastic constant *b* in units of [Nm/rad^2^]. When *b* is zero, the hexagonal structure is unstable and collapses upon shear or uniaxial stretch, while when *b* is very high the symmetric configuration of the hexagon strongly resists a change in shape. In any case, the walls each align and elongate in such a way that the total energy of the network is minimized at each strain step. In this way, the model of Cavalcante et al. (2005) takes into consideration the deformation energy stored within all the individual alveolar walls and all the bonds between their respective neighboring walls in a network.

In contrast, the analytical model developed above considers the tensile and bending energies of alveolar walls in isolation (i.e., it does not take into account how the presence of neighboring walls might affect a given wall's configuration). Both models, however, should predict the same total elastic energy in the tissue strip at each tissue strain. When their respective expressions for total energy are equated (see Appendix B for details), we find that

(4) $$\begin{array}{l} Y_{ae}=u\frac{9}{4}{\bar{l}}_{o}^{2}k \end{array}$$

(5) $$\begin{array}{l} Y_{be}=u18b \end{array}$$

where *l*_o_ is the mean initial length of the walls in the numerical model, and *u* is a unity constant that ensures both sides of Equations 4 and 5 have the same dimensions.

#### Experimental Model Validation

We tested the predictions of the analytical model against three different sets of experimental data. The first set comprised the stress-strain measurements made in excised rat lung slices (*n* = 5 per group) published by Cavalcante et al. (2005). Measurements were made while the strips were bathed in normal, hypertonic, and hypotonic saline in order to modulate the charge densities on the proteoglycans in the tissue, which affects tissue stiffness due to altered repulsive forces acting on alveolar walls. Because the surface charges on elastin and collagen are significantly lower than those on proteoglycans (Maroudas and Bannon, 1981; Chalmers et al., 1999), altering saline tonicity is expected to mainly affect *Y*_*be*_ with relatively little influence on *Y*_*ae*_.

The second test data set was comprised of quasi-static stress-strain curves measured in mice published by Shiwen et al. (2015). We used data from both wild-type mice (WT, *n* = 6) and from age-matched mice in which myocardin-related transcription factor-A (MRTF-A) had been knocked out (KO, *n* = 6). MRTF-A is known to lead to a reduction in type I collagen deposition, leading to tissue softening, and indeed these investigators showed that KO had reduced tissue stresses compared to WT. The reduction in stress was attributed to both a decrease in collagen content and a decrease in fibril diameter from 97 ± 24 nm in WT to 58 ± 9 nm in KO. It is thus expected that both *Y*_*f*_ and *Y*_*ae*_ should be reduced in the KO mice, with minimal alterations to *Y*_*be*_.

The third data set was collected in our laboratory in a study approved by the Institutional Animal Care and Use Committee of Boston University. We investigated the effects of aging on alveolar wall tensile and bond-bending moduli by fitting lung tissue stress-strain curves obtained in young (*n* = 5; 5 months) and old (*n* = 6; 19, and 24 months) mice. The young mice were obtained from Charles River Laboratories (Boston, MA). The mice were anesthetized by intraperitoneal injection of pentobarbital sodium (80 mg/kg), tracheostomized and cannulated with an 18-guage steel needle in the supine position followed by median abdominal incision and sternotomy under deep anesthesia. Euthanasia was obtained during exsanguination by cutting the abdominal aorta and inferior vena cava while the pulmonary artery was flushed with phosphate buffered saline (PBS). For the aged mice, isolated lungs were obtained from Brigham and Women's Hospital, Harvard Medical School. The left lung in each group was cut into strips with dimensions of 5 × 2 × 1 mm, and used to obtain tissue strip mechanics. Uniaxial quasi-static stress-strain curves were obtained as previously described (Cavalcante et al., 2005). Briefly, the system consists of a computer-controlled lever arm (Aurora Scientific, Ontario, Canada) and a sensitive force transducer (model 300B; Aurora Scientific, Ontario, Canada) both attached to an acrylic base housing a 24 mL tissue bath maintained at a temperature of 37°C. Small metal plates were glued to each end of the strip with cyanoacrylate. The plates were attached to the force transducer and lever-arm via steel wires and the strip was submerged in the tissue bath containing PBS. The strips were preconditioned by applying three consecutive triangular displacement signals peaking at 60% uniaxial macroscopic strain at a rate of 2 %/s. After a 5-min equilibration period, force-displacement data were collected. Stress and strain were calculated as the force divided by the initial cross-sectional area and the displacement divided by the initial length of the strip, respectively.

### Model Fitting and Statistical Analysis

Data simulation and curve fitting were performed using either MATLAB R2015a or R2018b (MathWorks, CA), while statistical tests were performed using SigmaPlot 11.0 (Systat Software, CA). A paired *t*-test was applied for the rat tissue slices bathed in different tonicities, while a *t*-test was used for the collagen knockout and aging study. Statistical significance was accepted at *p* < 0.05.

The *nlinfit* function in MATLAB was used to fit the model to data. The *nlinfit* solver was provided with initial guesses for *Y*_*ae*_ and *Y*_*be*_ and an initial uniform distribution of 5,000 alveolar wall angles between 0 and π/2 was assumed. The initial alveolar strains were set to zero. Since the model predictions (Equation 1) are based on the assumption of small incremental strains, while in most cases the experimental strain steps were large, we iteratively calculated the alveolar wall strains and angles as well as the total tissue stress for strains ranging from zero up to the maximum experimental strain, in strain steps of 0.001. The predicted model tissue stress was then compared to the experimental stress values at the experimental strains, the predicted values of *Y*_*ae*_ and *Y*_*be*_ were updated, and the process was repeated. Further investigations with the model indicated that at least 2,000 alveolar walls and an incremental strain of <0.01 are required for the model to converge—see Appendix C.

## Results

### Model Behavior

**Figure 2:**
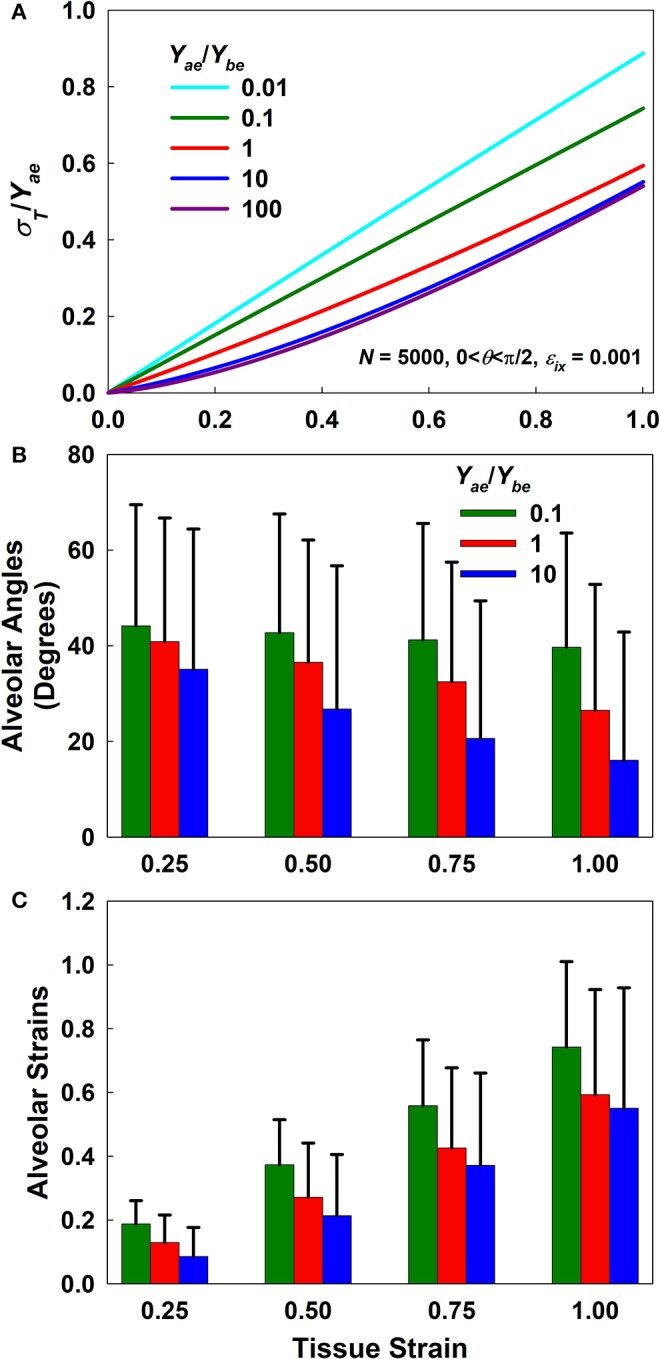
Forward simulation results. **(A)** Normalized stress-strain curves for different *Y*_*ae*_/*Y*_*be*_ ratios. **(B,C)** Mean and standard deviation of alveolar angles and strains, respectively, as a function of macroscopic tissue strain for *Y*_*ae*_/*Y*_*be*_ ratios of 0.1, 1, and 10. Alveolar angles at a strain of zero were uniformly distributed between 0 and π/2 while alveolar strains were set to zero.

Figure 2 plots the normalized stress-strain curves for five different *Y*_*ae*_/*Y*_*be*_ ratios (Figure 2A) as well as the means and standard deviations of the alveolar angles and strains for ratios of 0.1, 1, and 10 (Figures 2B,C, respectively). The normalized stress-strain relationship for a ratio of 0.01 is essentially linear (Figure 2A) while the mean wall angle of π/4 shows that alignment was minimal, demonstrating that strain was accommodated almost entirely through wall elongation. As *Y*_*ae*_/*Y*_*be*_ increases, however, the stress-strain relationships (Figure 2A) become progressively more non-linear and thus similar to the experimentally observed stress-strain curves, while the mean wall angles depart increasingly from π/4, indicative of an increasing degree of wall alignment with macroscopic stretch. Above a *Y*_*ae*_/*Y*_*be*_ ratio of 100 the macroscopic stretch of the tissue was accommodated almost entirely by wall alignment. Additional investigations of the effects of alveolar number and incremental strain are presented in Appendix C.

**Figure 3:**
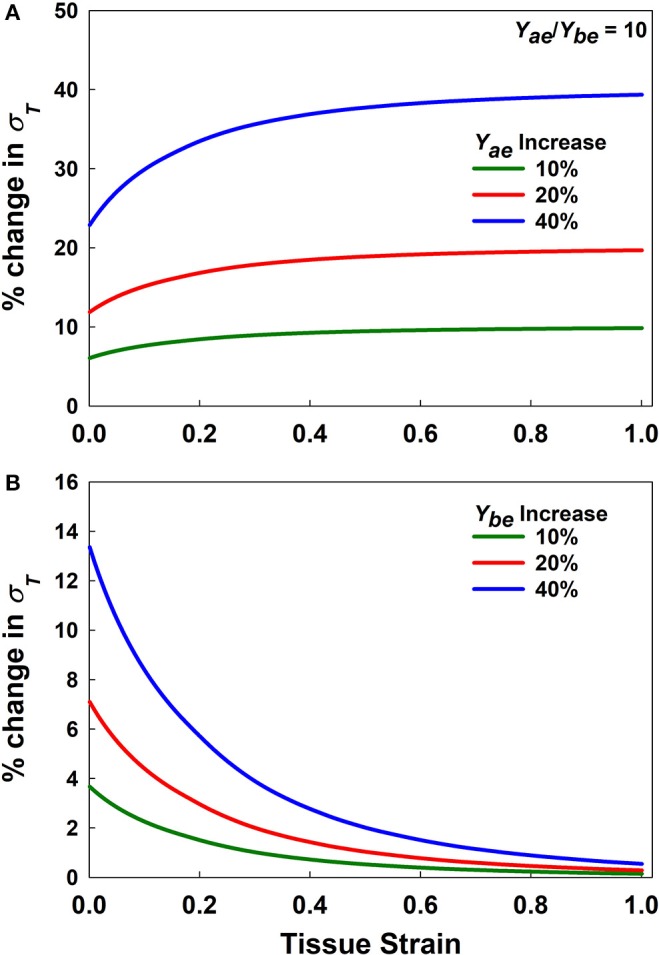
Sensitivity analysis. **(A,B)** % change in stress for increasing *Y*_*ae*_ and *Y*_*be*_, respectively by 10, 20, and 40% for *Y*_*ae*_/*Y*_*be*_ = 10.

Figure 3 presents a sensitivity analysis of the effects of variations in *Y*_*ae*_ and *Y*_*be*_ on model stress for a *Y*_*ae*_/*Y*_*be*_ ratio of 10. The effects of these two parameters are opposite; increasing *Y*_*ae*_ causes stress to increase (Figure 3A), while increasing *Y*_*be*_ causes stress to decrease (Figure 3B) with strain. Furthermore, variations in *Y*_*ae*_ have a stronger impact at high strains, while the opposite is true for *Y*_*be*_. Clearly, once strain is high enough to bring the alveolar walls into alignment, *Y*_*be*_ has no further effect on the stress-strain curve, so with further strain there is a linear relationship between *Y*_*ae*_ and stress. In contrast, a linear relationship between *Y*_*be*_ and stress is not possible. Figure 3 also shows that the rate of change of stress with either *Y*_*ae*_ or *Y*_*be*_ is greatest at low stress. This indicates that fitting the stress-strain relationship predicted by the model to experimental data over a range of strains from 0 to 30% should be sufficient to reliably estimate both elastic moduli.

### Model Validation

**Figure 4:**
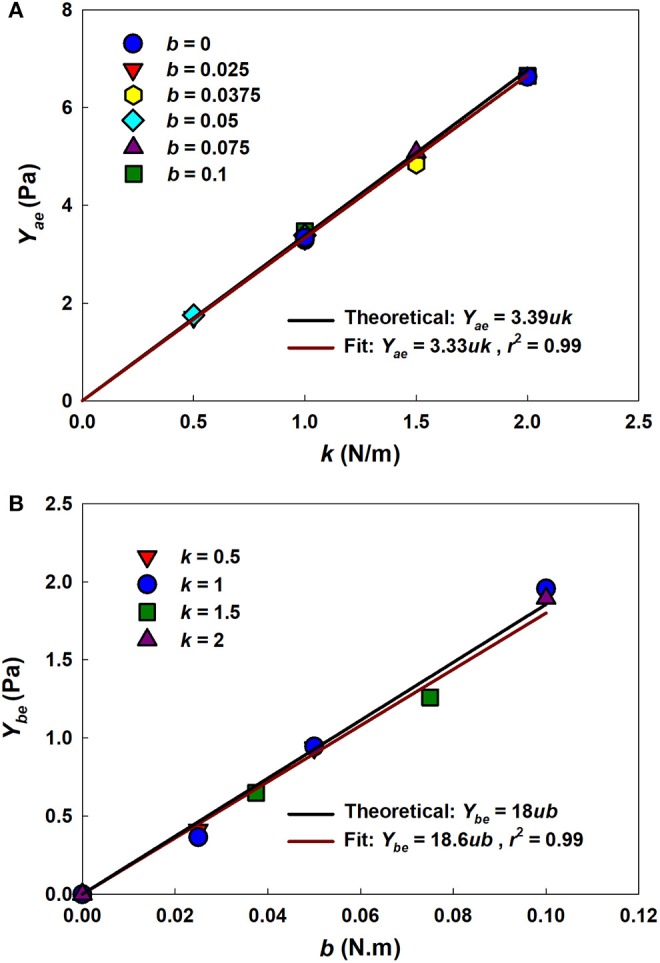
Uniaxial model-based estimates of network parameters. **(A)** *Y*_*ae*_ vs. *k* for different *b* values. **(B)** *Y*_*be*_ vs. *b* for different *k* values. Solid black lines represent the theoretical relationships between network and model parameters. Solid dark red lines represent the fits with the *r*^2^ as the adjusted coefficient of determination for the linear fit.

Figure 4 shows that estimates of *Y*_*ae*_ and *Y*_*be*_ from the analytical model (Equations 4 and 5, respectively) agree closely with those used in the numerical network model over a range of parameter values, with *r*^2^ values of 0.99. This supports the utility of the analytical model as a computationally much faster alternative to the numerical model.

**Figure 5:**
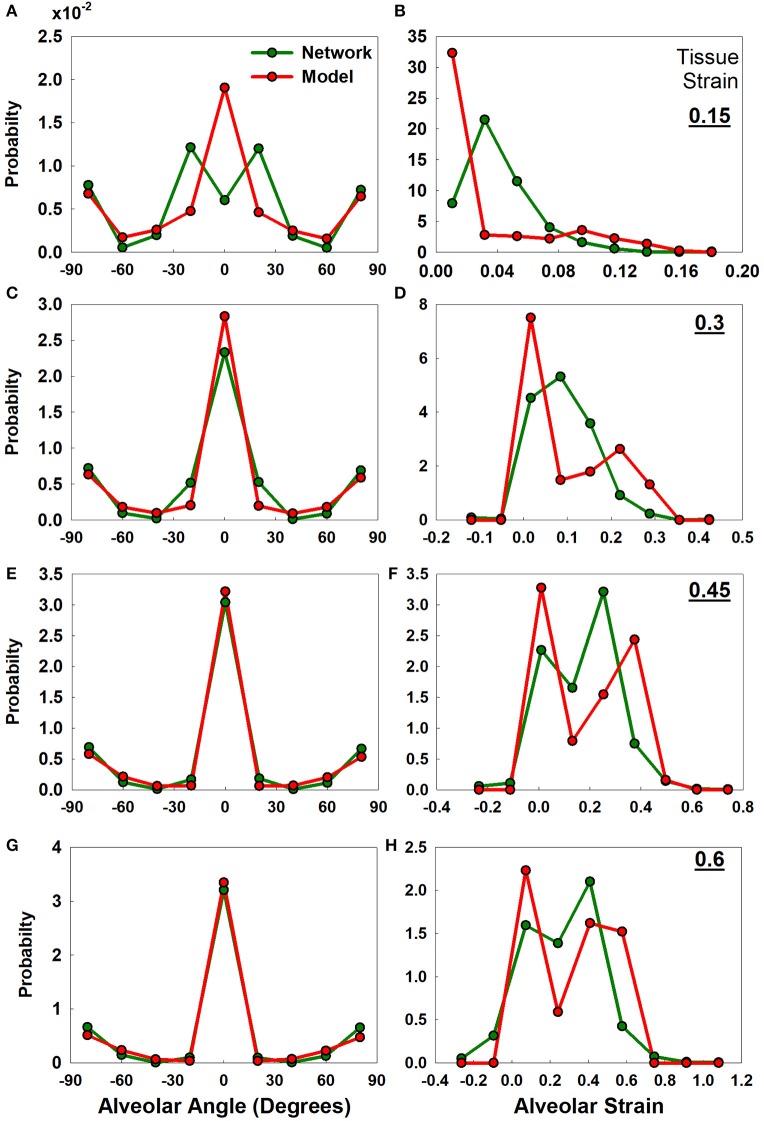
Uniaxial analytical and the network model-based alveolar angle and strain distributions. The graphs show alveolar angle probability distribution functions **(A,C,E,G)** and alveolar strain probability distribution functions **(B,D,F,H)** from the analytical model and the network at four levels of tissue strains (0.15, 0.3, 0.45, and 0.6). Initial alveolar strains are zero. Initial angle distribution is shown in Appendix B, Figure 9B.

The distributions of alveolar wall angle and wall strain at four different tissue strains for the analytical and numerical models are compared in Figure 5. There is generally good agreement between the two models in terms of wall angle (Figures 5A,C,E,G), although alveolar walls align with the direction of strain somewhat more rapidly in the analytical model because interactions between the nodes of the network model resist alignment to some degree. In particular, the analytical model assumes that the alveolar wall ends rotate according to the macroscopic strain; however, in the network model each node pulls on another wall which delays the alignment with strain. The strain distributions from the two models (Figures 5B,D,F,H) are less consistent than the angle distributions, although both models exhibit similar ranges of strain values. An important difference between the two models that may account for the discrepancies seen in the strain panels of Figure 5 is that while alveolar walls can only increase in length with macroscopic strain in the analytical model, in the network model it is possible for two adjacent nodes in the network model to approach each with increasing strain if the nodes are aligned perpendicular to the direction of strain (as illustrated in Figure 10B, Appendix B). The network model considers such shortened walls to be flaccid, thus making no contribution to the strain energy.

**Figure 6:**
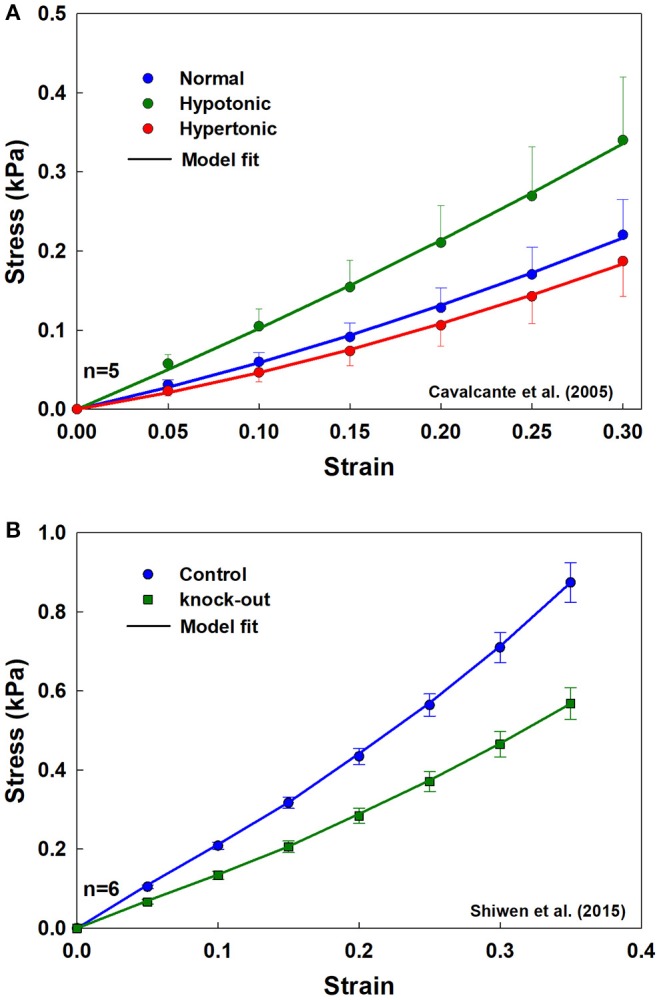
Population mean and standard error, with model fits (solid lines) for the tissue strips for **(A)** three different tonicities (normal, hypertonic, and hypotonic) from rat lungs, and **(B)** from WT and MRTF-A KO mice.

Figure 6 shows fits from the analytical model to stress-strain measurements in rat lung tissue strips made under different tonicity conditions (Figure 6A) and between WT and MRTF-A KO mice (Figure 6B). The fitted model parameters are shown in Table 1. Alterations in tonicity had no statistically significant effect on *Y*_*ae*_, but *Y*_*be*_ increased by 211% between normal and hypotonic conditions (*p* = 0.03) and showed a nearly significant (*p* = 0.09) decrease between normal and hypertonic conditions. On the other hand, *Y*_*ae*_ decreased significantly (*p* = 0.014) between WT and MRTF-A KO mice, while there was no significant change in *Y*_*be*_. These differential effects of changes in tonicity vs. MRTF-A KO are as expected on the basis of altered proteoglycan charge density vs. reduced collagen I deposition in the tissue.

**Table 1:** Model validation experimental results.

	***Y_***ae***_* (kPa)**	**%Δ**	***p*-value**	***Y_***be***_* (kPa)**	**%Δ**	***p*-value**
**Tonicity—Rat Tissue Strips**						
Normal	1.94 ± 1.07			0.27 ± 0.14		
Hypotonic	3.28 ± 1.75	69	0.11	0.84 ± 0.41	211	0.03
Hypertonic	1.78 ± 1.03	−8	0.11	0.11 ± 0.11	−60	0.09
**Collagen Knockout—Mouse Tissue Strips**						
Control	5.96 ± 1.39			1.28 ± 0.42		
Knock-out	3.94 ± 0.93	−34	0.014	0.84 ± 0.58	−34	0.165

*Y_ae_ and Y_be_ values are mean ± standard deviation. %Δ is the percent difference with respect to the normal control*.

**Figure 7:**
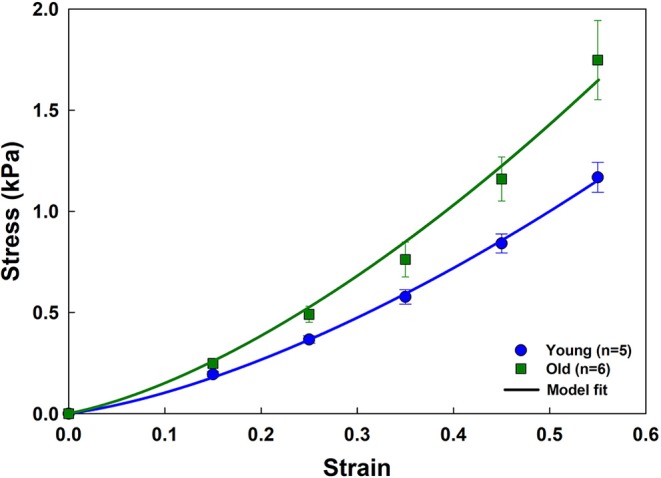
Stress-strain curves and parameter estimation results for the aging data. Experimental data (mean and standard errors) for young and old mice are shown with symbols. Model fits are plotted as solid lines.

Figure 7 shows that age increases parenchymal tissue stiffness, and that the analytical model recapitulates this effect. The elastic moduli obtained by fitting the model to the young and old stress-strain relationships are given in Table 2. Values for *Y*_*a*_ and *Y*_*b*_ as well as the fiber tensile elastic modulus (*Y*_*f*_) were estimated using literature values for the density of wall tissue in a parenchymal strip (δ_*V,w*_) and the density of fibers in an alveolar wall (δ_*V,f*_) obtained by Huang et al. (2007) in 2 and 20 month old C57BL/6 mice. They found that the tissue fraction was 35% independent of age, while the total fiber content (collagen plus elastin) decreased with age from 4 to 2.75%. The values of *Y*_*be*_ estimated by the analytical model did not change with age, but *Y*_*ae*_ increased by 34.4% from 4.4 to 5.9 kPa (*p* = 0.043). *Y*_*f*_ increased with age by 99.4% from 311 to 620 kPa (*p* = 0.001).

**Table 2:** Aging results showing mean and standard errors with respective *p*-values for young and old mice.

**Modulus (kPa)**	**Young (*n* = 5)**	**Old (*n* = 6)**	***p*-value**
*Y_*ae*_*	4.36 ± 0.45	5.86 ± 1.35	0.043
*Y_*be*_*	0.114 ± 0.001	0.161 ± 0.066	0.77
*Y_*a*_*	12.44 ± 1.14	15.49 ± 3.53	0.094
*Y_*b*_*	0.42 ± 0.25	0.46 ± 0.17	0.77
*Y_*f*_*	311 ± 29	620 ± 131	0.001

*Model fitted values for Y_ae_ and Y_be_ were used to derive Y_a_, Y_b_, and Y_f_. Y_a_ = average alveolar tensile elastic modulus; Y_b_ = average alveolar wall bond-bending elastic modulus; Y_f_ = average fiber tensile elastic modulus*.

## Discussion

In this study, we developed an analytical model of lung parenchymal tissue mechanics that links uniaxial stress-strain behavior at the level of the tissue strip to the alignment and elongation of alveolar walls. By modeling this behavior, we were able to separate and estimate the tensile and bond-bending moduli of alveolar walls under different experimental conditions and biological processes such as aging.

The model replicated experimental stress-strain data from a variety of sources and provided average estimates of the tensile modulus of an average alveolar wall and the bond-bending modulus of the junction between walls (Tables 1, 2). Investigations of model behavior (Figures 2, 3) suggest that the ratio of the tensile to the bond-bending elastic moduli must exceed 1 in order to account for the experimentally observed strain-stiffening properties of lung tissue (Fung, 1984; Karlinsky, 1992; Pinart et al., 2011; Suki and Bates, 2011; Yi et al., 2016; Polio et al., 2018). The stress-strain relationships predicted by the model become increasingly non-linear as the modulus ratio increases. This is consistent with the notion that the alveolar walls and the embedded fibers are the main load bearing elements in the lung parenchyma (Romero et al., 1998; Yuan et al., 2000; Maina and West, 2005; Yi et al., 2016), and is supported by our finding that reducing collagen in the wall reduces tissue stiffness (Figure 6B). Furthermore, since the elastic modulus of an alveolar wall in the model is constant with stretch, the non-linearity of the predicted stress-strain relationships arises purely from alignment that allows the walls to align with the direction of macroscopic strain, which is essentially a recruitment process. Alignment also implies a certain capacity of the wall to oppose being stretched, something that is modulated by the ratio of *Y*_*ae*_ to *Y*_*be*_. The higher the ratio, the stiffer the alveolar wall and the faster the alignment (Figure 2B). Ratios larger than 1 were obtained from the fitting (Tables 1, 2) which is expected on physiological grounds since the function of the wall, and particularly the fibers within it, is to provide a mechanically resilient structure within a very thin layer to support structural integrity and gas exchange (Weibel, 2009; Maina and West, 2005).

The estimated values of *Y*_*ae*_ (~5 kPa) and *Y*_*f*_ (~300 kPa for the young mice) obtained by fitting the analytical model to experimental stress-strain relationships match those reported in literature (Table 2). For example, Polio et al. (2018), measured *Y*_*ae*_ in porcine lung using cavitation rheology (6.1 ± 1.6 kPa), small amplitude oscillatory shear rheometry (3.3 ± 0.5), micro-indentation (1.4 ± 0.4), and uniaxial tension (3.4 ± 0.4) and showed that all these techniques provided values in the same range. Using AFM, Liu and Tschumperlin (2011) estimated a value around 5 kPa for mice, while Jorba et al. (2019) obtained values around 10 kPa at low strain in rats. Cavalcante et al. (2005) used their network model to estimate the effective alveolar wall stiffness to be ~5 kPa. Assuming that collagen fibers are the primary load bearing constituents of the wall, they also estimated fiber tensile elastic modulus to be ~300 kPa, which is similar to the average modulus in mice provided by our analytical model (Table 2) even though our estimate is an ensemble value including both elastin and collagen fibers. Furthermore, the elastic modulus of single elastin fibers is around 410 kPa (Aaron and Gosline, 1981). This is similar to the values of *Y*_*f*_ we obtained in mice in the present study (Table 2), perhaps because the delayed recruitment of flaccid collagen fibers causes elastin to be the primary determinant of stress at low strain (Mead, 1961).

The elastic moduli of the alveolar wall and the load-bearing fiber estimated by the analytical model both increased with age (Table 2), as did the stiffness of the tissue itself (Figure 7). Thus, even though the fiber content of the alveolar wall was reduced in the older mice, the fibers themselves were actually stiffer, something that can be attributed to the increased protein fiber cross-linking that is known to occur with age (Reiser et al., 1987; Snedeker and Gautieri, 2014). This implies that the increased lung compliance that occurs with age (Knudson et al., 1977; Suki and Bartolák-Suki, 2015) must be a reflection of age-related increase in airspace dimensions (Thurlbeck, 1967). More importantly, however, this finding also suggests that cells in the aging lung will experience an increased stiffening of the ECM substrate upon which they live, which has implications for cellular signaling and senescence (Suki and Bartolák-Suki, 2015).

Our analytical model thus recapitulates key experimental observations made in lung tissue strips and provides estimates of constitutive tissue properties that are consistent with experimental measurement and prediction by other models. These findings must nevertheless be viewed in light of the model limitations, which relate principally to the assumptions inherent in it. For example, a fundamental assumption of the model, made in the interests of mathematical tractability, is that alveolar walls can be represented as components that do not interact except insofar as to collaborate in the generation of bond-bending forces through their relative orientations. In other words, the orientation of a given wall is assumed not to influence the orientations of any of the neighboring walls to which it is connected. While this might seem open to question, the estimates of elastic modulus obtained by fitting the model to data generated by a numerical model compare well both to the parameters (Figure 4) and to wall angles and strains (Figure 5) in the numerical model even though the analytical model is not capable of recapitulating network effects that can take place in the numerical model.

Concerning validation by comparing the model to another model, we note the following. If an analytical model is developed with simplifying assumptions, it is often the case that such a model is compared to a more sophisticated numerical model that accounts for mechanisms that are necessarily omitted from the analytic approach. Since Cavalcante et al. (2005) did verify their model experimentally, we chose this model to investigate how network effects influenced the current model.

Another important simplifying assumption in the analytical model is that the stress-strain behavior of the alveolar wall is linear with a constant elastic modulus. A recent study used atomic force microscopy (AFM) to measure alveolar septal wall stiffness in decellularized rat lung tissue and found that indentation stiffness increased from about 10 kPa at 0 strain to about 80 kPa at 0.3 strain (Jorba et al., 2019). While the mechanism underlying this non-linear behavior remains unclear, one possibility is that it reflects the progressive straightening of flaccid fibers (Sobin et al., 1988; Mercer and Crapo, 1990; Toshima et al., 2004). Elastin fibers are generally presumed not to be flaccid under any conditions, so the flaccid fibers are probably collagen (Mercer and Crapo, 1990; Chen et al., 2013; Chow et al., 2014). Another possible explanation for the AFM results is geometric; the opposing force on a probe pushing perpendicular to a fiber under tension is due to the vector component of the tension in the fiber that is generated against the probe as the fiber bends. It is also possible that the stress-strain behavior of the fiber itself is actually non-linear. This behavior could be incorporated into the model at the expense of increased model complexity. As an example, the elastic moduli could be modeled using a polynomial equation to capture waviness or fiber stiffening. Even though this additional complexity might not have an analytical solution and thus would need to be solved numerically, the same geometrical considerations and energy minimization principles still hold and are applicable. Thus, in relation to the present study, it must be noted that the modulus parameters estimated by the analytical model represent averages of the tensile and bond-bending moduli of a large population of alveolar walls.

The analytical model also assumes affine kinematics, meaning that microstrains occurring in small parcels of tissue mirror the macrostrains of the tissue as a whole. This does not, however, apply to individual alveolar walls, which move according to their respective initial orientations. These orientations vary widely throughout the tissue, as they do in the numerical network model (Cavalcante et al., 2005), and also as found experimentally; alveolar wall segments violate the affine deformation assumption during uniaxial stretching (Brewer et al., 2003).

In summary, we have developed an analytical model that establishes a link between the mechanical behavior of lung tissue at the macroscopic tissue level and alveolar wall scale. Matching the model predictions to experimental data provides realistic values for the tensile and bond-bending moduli of individual alveolar walls, as well as their changes with age, collagen content, and matrix charge density. The model thus provides a potential platform upon which to understand how disease processes and aging affect lung function, and how interventions such as mechanical ventilation might be optimized to avoid damaging the tissue or avoid aberrant mechanotransduction of the cells contained within it.

## Data Availability Statement

The datasets generated for this study and the model as an executable file are available on request to the corresponding author.

## Ethics Statement

The animal study was reviewed and approved by Institutional Animal Care and Use Committee of Boston University.

## Author Contributions

SB, BS, and JB contributed to model development. AT collected data for the young and old mouse study. SB contributed to forward simulations and experimental data fitting. SB and BS contributed to data analysis. SB, BS, and JB contributed to writing the manuscript. All authors approved the submitted manuscript.

### Conflict of Interest

The authors declare that the research was conducted in the absence of any commercial or financial relationships that could be construed as a potential conflict of interest.
