# Supplementary material for: An Analytical Model for Estimating Alveolar Wall Elastic Moduli From Lung Tissue Uniaxial Stress-Strain Curves
Source: Front Physiol. 2020 Feb 25;11:121. doi: 10.3389/fphys.2020.00121 (PMC7052331; doi:10.3389/fphys.2020.00121)
Supplement: Supplementary file 1 [file Presentation_1.pdf]

Appendix A: Model derivation

We assume that an alveolar wall has tensile elastic modulus Y_a and bond-bending elastic modulus Y_b, and is embedded in a lung tissue strip with initial length X_o (Figure 8). The alveolar wall has initial length l_o, and is aligned at initial angle θ_o with respect to the horizontal axis. The tissue is stretched from this initial configuration in a series of incremental length increases $dX_s \ll 1$, $s = 1,2,3 \ldots$. We assume that the alveolar wall remains straight, so a rectangular block of lung tissue exists that includes the alveolar wall as its diagonal. The horizontal dimension of this tissue block (i.e., the dimension in the direction of stretch) is x_s, and this dimension increases by dx_s with each incremental increase in tissue length. Assuming affine transformations between micro and macro scales in the tissue, the strain on the tissue block equals the strain on the whole tissue (Figure 8A). This does not apply to the alveolar wall itself, however, because the wall is free to both rotate and stretch with tissue strain (Figure 8B). The initial length of the wall and the initial horizontal dimension of the tissue block ($x_{o,s}$) at stretch step s are thus related by $x_{o,s} = l_o \cos\theta_s$ (Figure 8A).

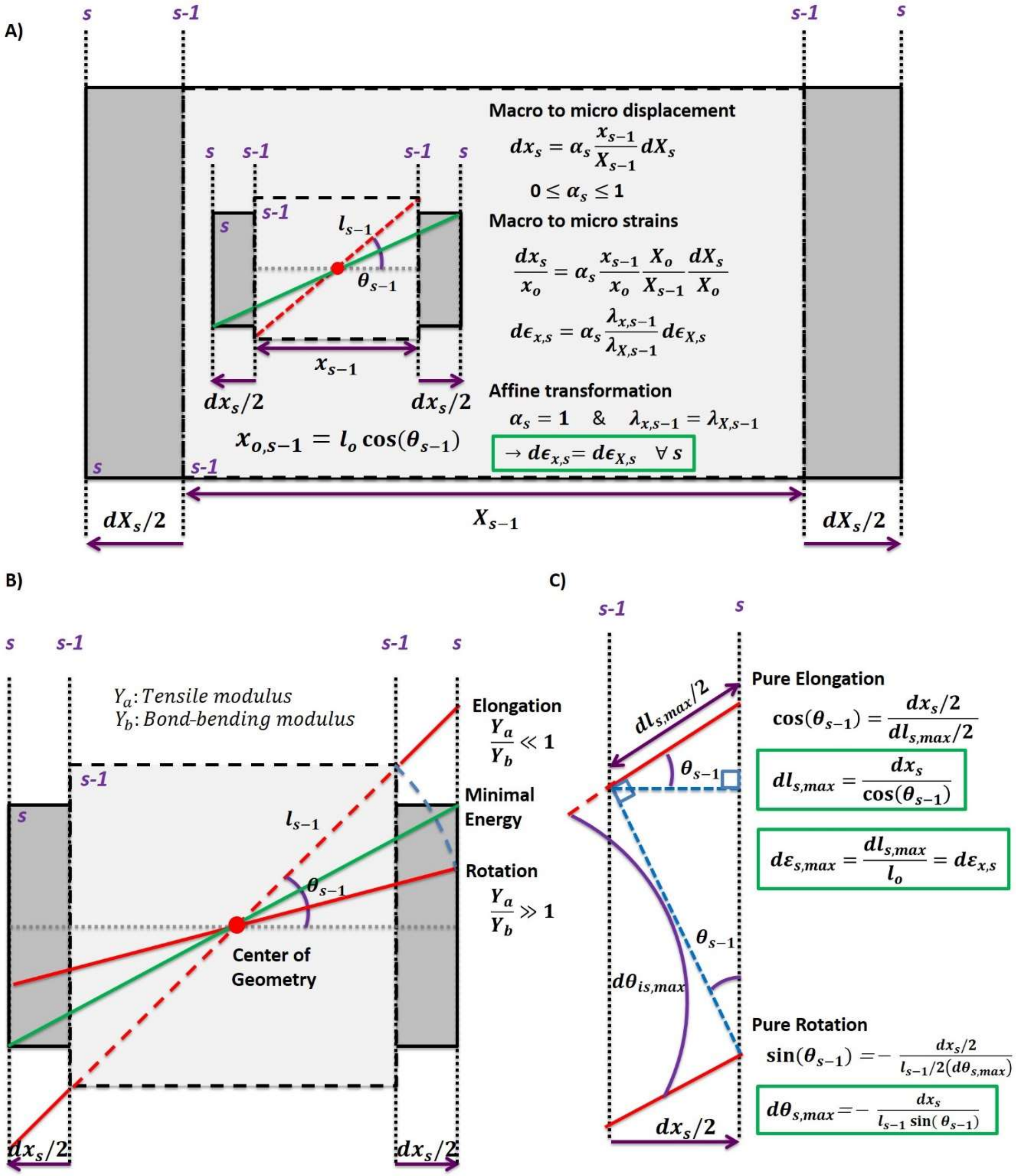

Figure 1: Uniaxial model illustration. A) A microscopic rectangular block of tissue is defined so as to confine an alveolar wall segment along its diagonal. A macroscopic horizontal displacement dX_s causes a microscopic displacement dx_s. The incremental microscopic and macroscopic strains are equal when $\alpha_s = 1$), while the microscopic strain is zero when $\alpha_s = 0$. We assume that the tissue block follows an affine transformation such that a microscale tissue block follows the macroscale deformation. This does not apply to a single alveolar wall, however, because it has the ability to rotate. B) An alveolar wall (dashed red line) with angle θ_{s-1} and length l_{s-1} is embedded within the microscopic tissue block which is stretched by a microscopic displacement dx_s. The wall has two extreme points governed by the ratio of tensile to bond-bending elastic moduli. If the ratio is much smaller than 1, the wall will elongate symmetrically from its center point without rotation, but if the ratio is much larger than 1 the wall will rotate about its center point without elongation. The ends of the wall in its new configuration (green solid line) lie at points that minimize the total wall energy on the vertical line connecting the two extreme points corresponding to either pure rotation or pure

elongation. C) For small displacements, the maximum rotation and elongation are a linear function of dx.

The changes in wall length (dl_s) and angle ($d\theta_s$) between stretch steps $s-1$ and s are (Figure 8B):

$$dl_s = l_s - l_{s-1} \tag{A.1}$$

$$d\theta_s = \theta_s - \theta_{s-1} \tag{A.2}$$

The new configuration of the wall after stretch, which minimizes the elastic potential energy of the system, lies between two extremes corresponding to either pure elongation or pure rotation (Figure 8B). When $\frac{Y_a}{Y_b} \ll 1$, the wall will only elongate to minimize energy, leading to:

$$d\theta_{s,min} = 0 \tag{A.3}$$

and

$$dl_{s,max} = \frac{dx_s}{\cos(\theta_{s-1})} \tag{A.4}$$

When $\frac{Y_a}{Y_b} \gg 1$, the wall will only rotate to minimize energy, in which case

$$d\theta_{s,max} = \cos^{-1}\left(\frac{l_{s-1}cos(\theta_{s-1}) + dx_s}{l_{s-1}}\right) - \theta_{s-1} \tag{A.5}$$

and

$$dl_{s,min} = 0 \tag{A.6}$$

Most likely, the ends of the wall in its new configuration will lie somewhere along the vertical line bordered by the two extremes (Figure 8B). Assuming small displacements and maintaining a positive slope, the relationship between the incremental changes in length and angle are linearly related to each other thus:

$$dl_s = dl_{s,max} - \frac{dl_{s,max}}{d\theta_{s,max}} d\theta_s \tag{A.7}$$

Since changes in angle are small (Figure 8B), we can write:

$$\tan(\theta_{s-1}) \cong -\frac{dl_{s,max}}{l_{s-1}d\theta_{s,max}} \tag{A.8}$$

which means:

$$-\frac{dl_{s,max}}{d\theta_{s,max}} \cong l_{s-1}\tan(\theta_{s-1}) \tag{A.9}$$

This gives:

$$dl_s \cong l_{s-1}\tan(\theta_{s-1})\,d\theta_s + \ dl_{s,max} = l_{s-1}\tan(\theta_{s-1})\,d\theta_s + \frac{dx_s}{\cos(\theta_{s-1})} \tag{A.10}$$

Dividing by the initial length of the wall (l_o) gives the incremental strain as:

$$d\varepsilon_s \cong (1+\varepsilon_{s-1})\tan(\theta_{s-1})\,d\theta_s + d\epsilon_{x,s} \tag{A.11}$$

where

$$d\varepsilon_s = \frac{dl_s}{l_o} = \frac{l_s - l_{s-1}}{l_o} \tag{A.12}$$

and ε_s is the total wall strain at step s, defined as:

$$\varepsilon_s = \frac{l_s - l_o}{l_o} = \frac{l_s - l_{s-1}}{l_o} + \frac{l_{s-1} - l_o}{l_o} = d\varepsilon_s + \varepsilon_{s-1} \tag{A.13}$$

The incremental microscopic strain of the tissue block in the direction of stretch is:

$$d\epsilon_{x,s} = \frac{dx_s}{l_o\cos(\theta_{s-1})} = \frac{dx_s}{x_{o,s-1}} \tag{A.14}$$

which is assumed to be equal to the incremental macrostrain ($d\epsilon_{X,s}$) for affine deformations. Let p_1 and p_2 be the slope and y-intercept of the relation between $\mathrm{d}l_s$ and $d\theta_s$, respectively, given by:

$$p_1 = (1+\varepsilon_{s-1})\tan(\theta_{s-1}) \tag{A.15}$$

and

$$p_2 = \ d\epsilon_{x,s} \tag{A.16}$$

so that

$$d\varepsilon_s = p_1\, d\theta_s + p_2 \tag{A.17}$$

For a linearly elastic wall with a tensile spring constant k and a bond-bending spring constant b, the elastic energy, E_s, at a given stretch is:

$$E_s = \frac{k(l_s - l_o)^2}{2} + \frac{b(\theta_s - \theta_o)^2}{2} \tag{A.18}$$

The energy density, e_s, of the wall is:

$$e_s = \frac{E_s}{V_s} = \frac{k(l_s - l_o)^2}{2A_s l_s} + \frac{b(\theta_s - \theta_o)^2}{2V_s} \tag{A.19}$$

where A_s is the cross-sectional area of the wall and V_s is wall volume. If A_o is the initial cross-sectional area of the wall and V_o is its initial volume, and the wall material is incompressible, conservation of mass implies that $V_s = V_o = A_s l_s = A_o l_o$. Equation A.19 can then be written as:

$$e_s = \frac{Y_a(\varepsilon_s)^2}{2} + \frac{Y_b(\theta_s - \theta_o)^2}{2} \tag{A.20}$$

where Y_a and Y_b are the tensile and bond-bending elastic moduli of the wall defined, respectively, as:

$$Y_a = \frac{k l_o}{A_o} \tag{A.21}$$

and

$$Y_b = \frac{b}{V_o} \tag{A.22}$$

If $d\varepsilon_s^*$ and $d\theta_s^*$ are the incremental strain and angle, respectively, that minimize the total elastic energy of the wall, Eq. A.20, can be expressed as:

$$e_s = \frac{Y_a(d\varepsilon_s^* + \varepsilon_{s-1})^2}{2} + \frac{Y_b(d\theta_s^* + \Delta\theta_{s-1})^2}{2} \tag{A.23}$$

where $\Delta\theta_{s-1} = \theta_{s-1} - \theta_o$ is the total change in angle at step $s-1$. Equation A.23 can be written as:

$$e_s = e_{s-1} + \frac{Y_a (d\varepsilon_s^*)^2}{2} + \frac{Y_b (d\theta_s^*)^2}{2} + Y_a d\varepsilon_s^* \varepsilon_{s-1} + Y_b d\theta_s^* \Delta\theta_{s-1}$$
$$= e_{s-1} + \frac{Y_a (d\varepsilon_s^*)^2}{2} + \frac{Y_b \left(\frac{d\varepsilon_s^* - p_2}{p_1}\right)^2}{2} + Y_a d\varepsilon_s^* \varepsilon_{s-1} + Y_b \left(\frac{d\varepsilon_s^* - p_2}{p_1}\right) \Delta\theta_{s-1} \quad \text{(A.24)}$$
$$= e_{s-1} + \frac{Y_a (d\varepsilon_s^*)^2}{2} + \frac{Y_b \left(\frac{d\varepsilon_s^* - p_2}{p_1}\right)^2}{2} + Y_a d\varepsilon_s^* \varepsilon_{s-1} + Y_b \left(\frac{d\varepsilon_s^* - p_2}{p_1}\right) \Delta\theta_{s-1}$$

The position of the wall at s minimizes the energy density and it can be found by setting the derivative of the energy density with respect to incremental strain to zero:

$$\frac{de_s}{d(d\varepsilon_s^*)} = 0 = Y_a (d\varepsilon_s^*) + Y_b \left(\frac{d\varepsilon_s^* - p_2}{p_1^2}\right) + Y_a \varepsilon_{s-1} + \frac{Y_b \Delta\theta_{s-1}}{p_1} \quad \text{(A.25)}$$

Therefore,

$$d\varepsilon_s^* = \frac{p_2 - p_1^2 \frac{Y_a}{Y_b} \varepsilon_{s-1} - \Delta\theta_{s-1} p_1}{1 + \frac{Y_a}{Y_b} p_1^2}$$
$$= \frac{d\epsilon_{x,s} - \frac{Y_a}{Y_b} (1 + \varepsilon_{s-1})^2 \varepsilon_{s-1} \tan^2(\theta_{s-1}) - (1 + \varepsilon_{s-1}) \Delta\theta_{s-1} \tan(\theta_{s-1})}{1 + \frac{Y_a}{Y_b} (1 + \varepsilon_{s-1})^2 \tan^2(\theta_{s-1})} \quad \text{(A.26)}$$

Note that p_2 is a differential quantity (Eq. A16). From Eq. A.17 we obtain:

$$d\theta_s^* = \frac{d\varepsilon_s^* - p_2}{p_1} = -\frac{\frac{Y_a}{Y_b} p_1 p_2 + \frac{Y_a}{Y_b} \varepsilon_{s-1} p_1 + \Delta\theta_{s-1}}{1 + \frac{Y_a}{Y_b} p_1^2}$$
$$= -\frac{\frac{Y_a}{Y_b} \left[d\epsilon_{x,s} + \varepsilon_{s-1}\right] (1 + \varepsilon_{s-1}) \tan(\theta_{s-1}) + \Delta\theta_{s-1}}{1 + \frac{Y_a}{Y_b} (1 + \varepsilon_{s-1})^2 \tan^2(\theta_{s-1})} \quad \text{(A.27)}$$

thus the energy equation A.24 for a single alveolar wall becomes:

$$
\begin{aligned}
e_s &= e_{s-1} + \frac{Y_a}{2}\left[\frac{p_2^2}{1+\frac{Y_a}{Y_b}p_1^2}\right]\left[1 + 2\frac{\varepsilon_{s-1}}{p_2} - 2\Delta\theta_{s-1}\frac{p_1}{p_2} \right. \\
&\quad \left. - \frac{1}{p_2^2}\frac{Y_a}{Y_b}\left(\varepsilon_{s-1}p_1 + \frac{Y_b}{Y_a}\Delta\theta_{s-1}\right)^2\right] \\
&= e_{s-1} \\
&\quad + \frac{Y_a}{2}\left[\frac{1}{1+\frac{Y_a}{Y_b}(1+\varepsilon_{s-1})^2 tan^2(\theta_{s-1})}\right]\left[d\epsilon_{x,s}^2 \right. \\
&\quad + 2d\epsilon_{x,s}[\varepsilon_{s-1} - (1+\varepsilon_{s-1})\tan(\theta_{s-1})\Delta\theta_{s-1}] \\
&\quad \left. - \frac{Y_a}{Y_b}\left(\varepsilon_{s-1}(1+\varepsilon_{s-1})\tan(\theta_{s-1}) + \frac{Y_b}{Y_a}\Delta\theta_{s-1}\right)^2\right]
\end{aligned} \tag{A.28}
$$

This can be written in terms of effective alveolar strain ($\varepsilon_{s,eff}$) and effective alveolar stiffness ($Y_{eff,s}$) as:

$$
e_s = e_{s-1} + \frac{1}{2}Y_{eff,s}\varepsilon_{s,eff}^2 \tag{A.29}
$$

where the effective alveolar strain $\varepsilon_{s,eff}$ is related to the alveolar wall strain, alveolar angle, and incremental macroscopic strain by:

$$
\begin{aligned}
\varepsilon_{s,eff}^2 = &\left[d\epsilon_{x,s} + \varepsilon_{s-1} - (1+\varepsilon_{s-1})\tan(\theta_{s-1})\Delta\theta_{s-1}\right]^2 \\
&- [\varepsilon_{s-1} - (1+\varepsilon_{s-1})\tan(\theta_{s-1})\,\Delta\theta_{s-1}]^2 \\
&- \left[\sqrt{\frac{Y_a}{Y_b}}\varepsilon_{s-1}(1+\varepsilon_{s-1})\tan(\theta_{s-1}) + \sqrt{\frac{Y_b}{Y_a}}\Delta\theta_{s-1}\right]^2
\end{aligned} \tag{A.30}
$$

and the effective wall modulus is:

$$
Y_{eff,s} = \frac{Y_a}{1+\frac{Y_a}{Y_b}(1+\varepsilon_{s-1})^2\tan^2(\theta_{s-1})} \tag{A.31}
$$

The stress on an individual wall (σ_a) in the direction of stretch is:

$$
\sigma_a(s) = \frac{de_s}{d(d\epsilon_{x,s})} = Y_{eff,s}\left[d\epsilon_{x,s} + \varepsilon_{s-1} - (1+\varepsilon_{s-1})\tan(\theta_{s-1})\Delta\theta_{s-1}\right] \tag{A.32}
$$

Finally, the total energy of the tissue is calculated by summing the energy of all walls:

$$E_T = \sum_{n=1}^{N} E_{n,s} \tag{A.33}$$

giving the total tissue stress as:

$$\begin{aligned}\sigma_T(s) &= \frac{d}{d(d\epsilon_{x,s})}\left(\frac{E_T(s)}{V_T}\right) = \sum_{n=1}^{N} \frac{1}{V_T}\frac{dE_{n,s}}{d(d\epsilon_{x,s})} = \sum_{f=n}^{N} \frac{V_o}{V_T}\sigma_a(s) \\ &= \sum_{n=1}^{N} \delta_{n,a} Y_{n,a} \alpha_{n,s-1}\left[d\epsilon_{x,s} + \ \varepsilon_{n,s-1}\right. \\ &\quad \left. + (1 + \varepsilon_{n,s-1})(\theta_{n,i} - \theta_{n,s-1})\tan(\theta_{n,s-1})\right]\end{aligned} \tag{A.34}$$

where δ_a is the volume fraction of a single alveolar wall and V_T is the total tissue volume. Notice that the subscript n has been included here to denote the nth wall segment in the tissue. We can simplify this expression by replacing individual alveolar volume fraction and stiffness in the summation with their average value and defining δ_T as the total volume fraction of the alveolar walls in the tissue. That is:

$$\delta_T = N\delta_a \tag{A.35}$$

so

$$\sigma_T(s) = \delta_T Y_a \frac{1}{N}\sum_{n=1}^{N} \frac{d\epsilon_{x,s} + \ \varepsilon_{n,s-1} + (1 + \varepsilon_{n,s-1})(\theta_{n,o} - \theta_{n,s-1})\tan(\theta_{n,s-1})}{1 + \frac{Y_a}{Y_b}(1 + \varepsilon_{s-1})^2\tan^2(\theta_{s-1})} \tag{A.36}$$

Furthermore, assuming the effective tensile and bond-bending elastic moduli are:

$$Y_{ae} = \delta_T Y_a \tag{A.37}$$

and

$$Y_{be} = \delta_T Y_b \tag{A.38}$$

The final expression of stress can thus be written as:

$$\sigma_T(s) = \frac{Y_{ae}}{N}\sum_{n=1}^{N} \frac{d\epsilon_{x,s} + \varepsilon_{n,s-1} + (1 + \varepsilon_{n,s-1})(\theta_{n,o} - \theta_{n,s-1})\tan(\theta_{n,s-1})}{1 + \frac{Y_{ae}}{Y_{be}}(1 + \varepsilon_{s-1})^2\tan^2(\theta_{s-1})} \tag{A.39}$$

Note that the incremental strain and angle of an individual alveolar wall are functions of the elastic modulus ratio values, so they can be expressed in terms of the ratio of either the absolute or effective elastic moduli. The model can thus be fit to experimental stress-strain data in order to estimate the effective modulus values. In addition, knowing the total wall volume fraction composed of elastic fibers, the absolute elastic modulus values can also be estimated. Finally, the tissue's incremental modulus (Y_T) is:

$$Y_T(s) = \frac{d\sigma_T}{d(d\epsilon_{x,s})} = \frac{1}{N}\sum_{n=1}^{N} Y_{eff,s} = \frac{1}{N}\sum_{n=1}^{N} \frac{Y_a}{1 + \frac{Y_a}{Y_b}(1+\varepsilon_{s-1})^2 \tan^2(\theta_{s-1})} \tag{A.40}$$

Appendix B: Comparison of the analytical and numerical network models

The numerical model of Cavalcante et al. (2005) represents the alveoli in cross-section as a hexagonal network, where each side of the hexagon corresponds to an alveolar wall segment. Initially, a network of equal sided hexagons is created, and then the wall angles are perturbed randomly in order to form a heterogeneous network (Figure 9).

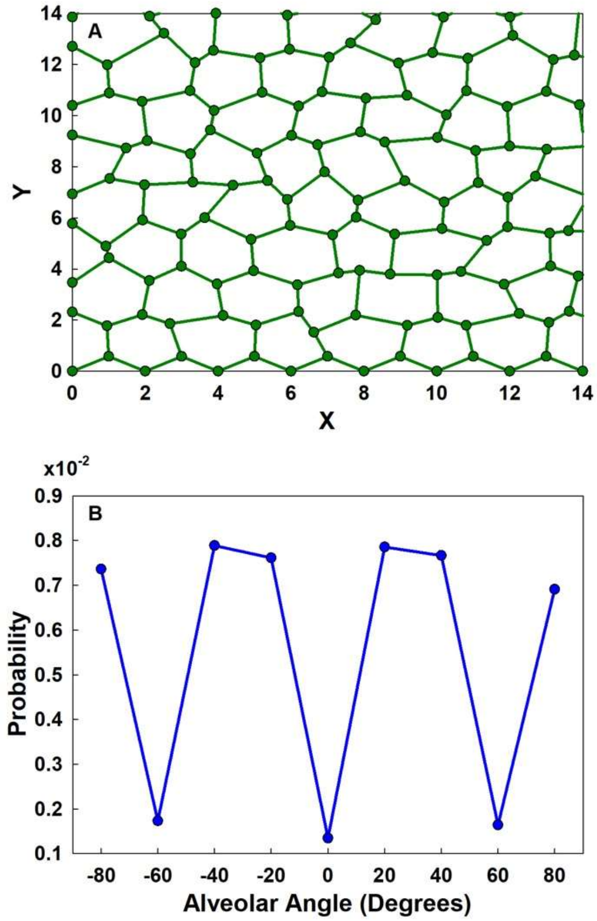

Figure 9: Hexagonal network. A) Selected 14 x 14 alveolar region from a 180 x 30 network. B) Probability density distribution function of the initial alveolar angles.

Each alveolar wall is represented as a spring with spring constant (k) and initial length (l_o), and the rotation of alveolar walls with respect to each other is elastically hindered by a bond-bending constant (b) such that the spring energy and the rotational energy are, respectively,

$$E_k = \frac{k l_o^2 (\varepsilon)^2}{2} \tag{B.1}$$

and

$$E_b = \frac{b(\Delta\theta)^2}{2} \tag{B.2}$$

where ε is the strain on the spring and $\Delta\theta$ is the change in angle between two neighboring alveolar walls. The total network energy (E_{nT}) is

$$E_{nT} = \frac{1}{2} k l_o^2 \sum_{j=1}^{Springs} \varepsilon_j^2 + \frac{1}{2} b \sum_{i=1}^{Nodes} (\Delta\theta_j)^2 \tag{B.3}$$

At each tissue strain, the total network energy is minimized to find the new configuration of all the alveolar walls (as opposed to minimizing the energy of a single alveolar wall as in the analytical model described above). Thus, unlike the analytical model, the network model includes wall-wall interactions and does not assume affine transformations.

The number of springs in the network model is N_s, while the volume of the network is defined as the number of hexagons N_h. For large networks, the number of springs per hexagon (N_s/N_h) is equal to 3 (2005). In order to compare the network model and the analytical model, we set $N_s = N$ and $N_h = uV_T$, where u is a unity constant with dimensions that make the dimensionality of the two models consistent. The mean initial length of the alveolar walls in the model is $\bar{l}_o$.

In order to find the equation relating k to Y_{ae} and b to Y_{be}, we equate the energies of a single hexagon in the numerical network model to that in the analytical uniaxial model. Consider a network formed by a homogenous pattern of regular equal-sided hexagons (Figure 10). Assume that the hexagonal network is stretched uniaxially perpendicular to two parallel sides of the hexagons. Thus, for all hexagons, these two sides will remain parallel to the direction of stretch but the remaining four sides will change their angles by $\Delta\theta$ (Figure 10D).

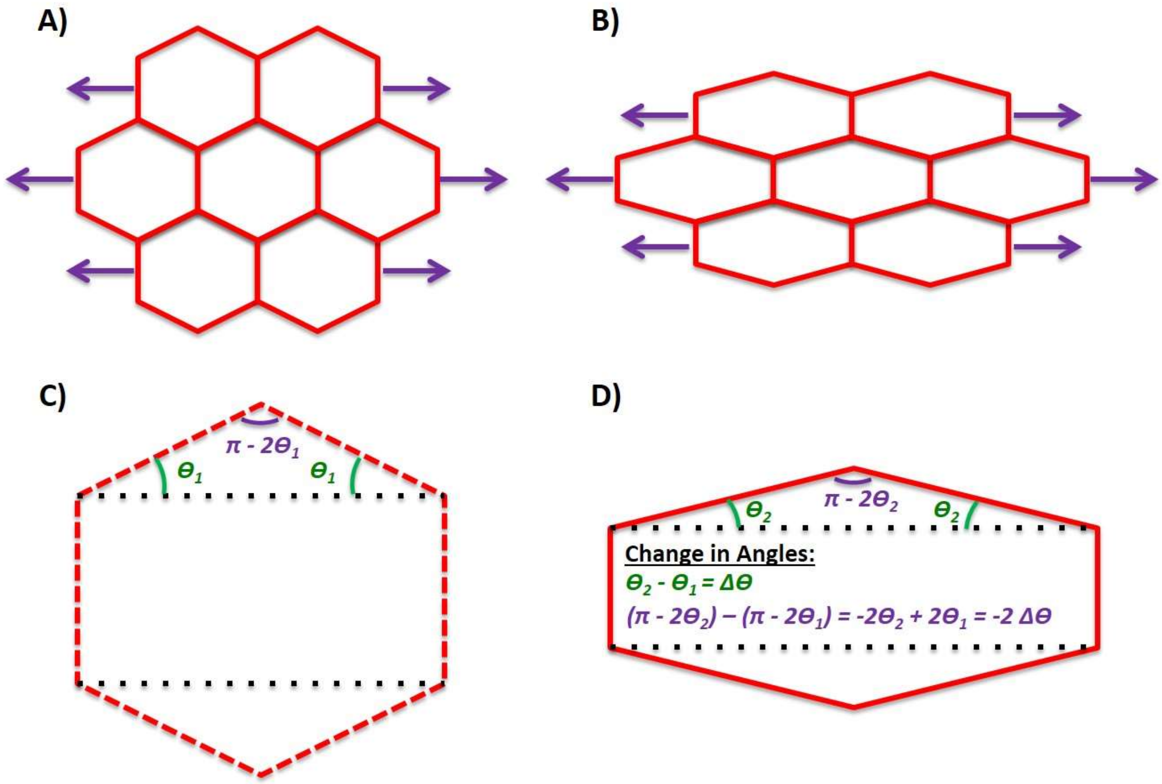

Figure 10: Hexagonal model under stretching. A) A pattern of homogeneous hexagons in the unstretched state. B) During stretch, the hexagons contract laterally as 4 out of 6 sides rotate inward and stretch while the other two become compressed without rotation. C) A hexagon before stretch has an acute angle of θ_1 between a rotating side and the direction of stretch. The obtuse angle between the two rotating sides is equal to $\pi - 2\theta_1$. D) After stretch these two angles become θ_2 and $\pi - 2\theta_2$, respectively. The corresponding changes in angle are $\Delta\theta = (\theta_2 - \theta_1)$ and $-2\Delta\theta$, respectively.

In order to find a relationship between k and Y_{ae}, we note that the strains are defined similarly in both models, and the alveolar walls in the network model bear no stress in compression. The contribution to the elastic energy, $E_{nh,k}$, of a typical hexagon of alveolar walls within the network is given by the product of the number of springs per hexagon (N_s/N_h) and the energy of a single wall, which is

$$E_{nh,k} = \frac{N_s}{N_h}\frac{k\bar{l}_o^2(\varepsilon_s)^2}{2} \tag{B.4}$$

Since only 4 out of 6 walls per hexagon become stretched, the corresponding energy $E_{ah,k}$ for the analytical model is

$$E_{ah,k} = 4\frac{Y_{ae}V_T(\varepsilon_s)^2}{2N} = 4\frac{N_h}{uN_s}\frac{Y_{ae}(\varepsilon_s)^2}{2} \tag{B.5}$$

Equating both energies gives

$$4\frac{N_h}{uN_s}\frac{Y_{ae}(\varepsilon_s)^2}{2}=\frac{N_s}{N_h}\frac{k\bar{l}_o^2(\varepsilon_s)^2}{2} \tag{B.6}$$

and hence

$$Y_{ae}=u\left(\frac{N_s}{N_h}\right)^2\frac{1}{4}\bar{l}_o^2k\cong u\frac{9}{4}\bar{l}_o^2k \tag{B.7}$$

To obtain a relationship between b and Y_{be}, we note that the network applies the rotational bond-bending energy at each node and thus has a different definition for $\Delta\theta$ than the analytic model (Figure 10). The total rotational energy $E_{nh,b}$ of a hexagon in the network is

$$E_{nh,b}=\left[4\frac{1}{2}b\Delta\theta^2+2\frac{1}{2}b(2\Delta\theta)^2\right]=6b\Delta\theta^2 \tag{B.8}$$

The changes in angles are analyzed in Figure 10C and 10D. Since only 4 out of 6 walls rotate within each hexagon, the corresponding bond-bending energy ($E_{ah,b}$) of the analytical model is written as

$$E_{ah,b}=\left(\frac{4}{6}\frac{N_s}{N_h}\right)\frac{1}{2}\frac{Y_{be}V_T}{N}\Delta\theta^2=\frac{\Delta\theta^2}{3u}Y_{be} \tag{B.9}$$

Equating both energies gives

$$\frac{\Delta\theta^2}{3u}Y_{be}=6b\Delta\theta^2 \tag{B.10}$$

hence

$$Y_{be}=u18b \tag{B.11}$$

Note that although we used a specific example to calculate the relationships expressed in Eqs. B.7 and B.11, the parameters k, b, Y_{ae}, and Y_{be} are material properties independent of geometry. Therefore, these relationships are valid in all cases.

Appendix C: Additional model simulations

Simulation results demonstrated that the numerical solution converged when the alveolar number exceeded 500 (Fig. 11A) and the incremental strain exceeded 0.01 (Fig. 11B).

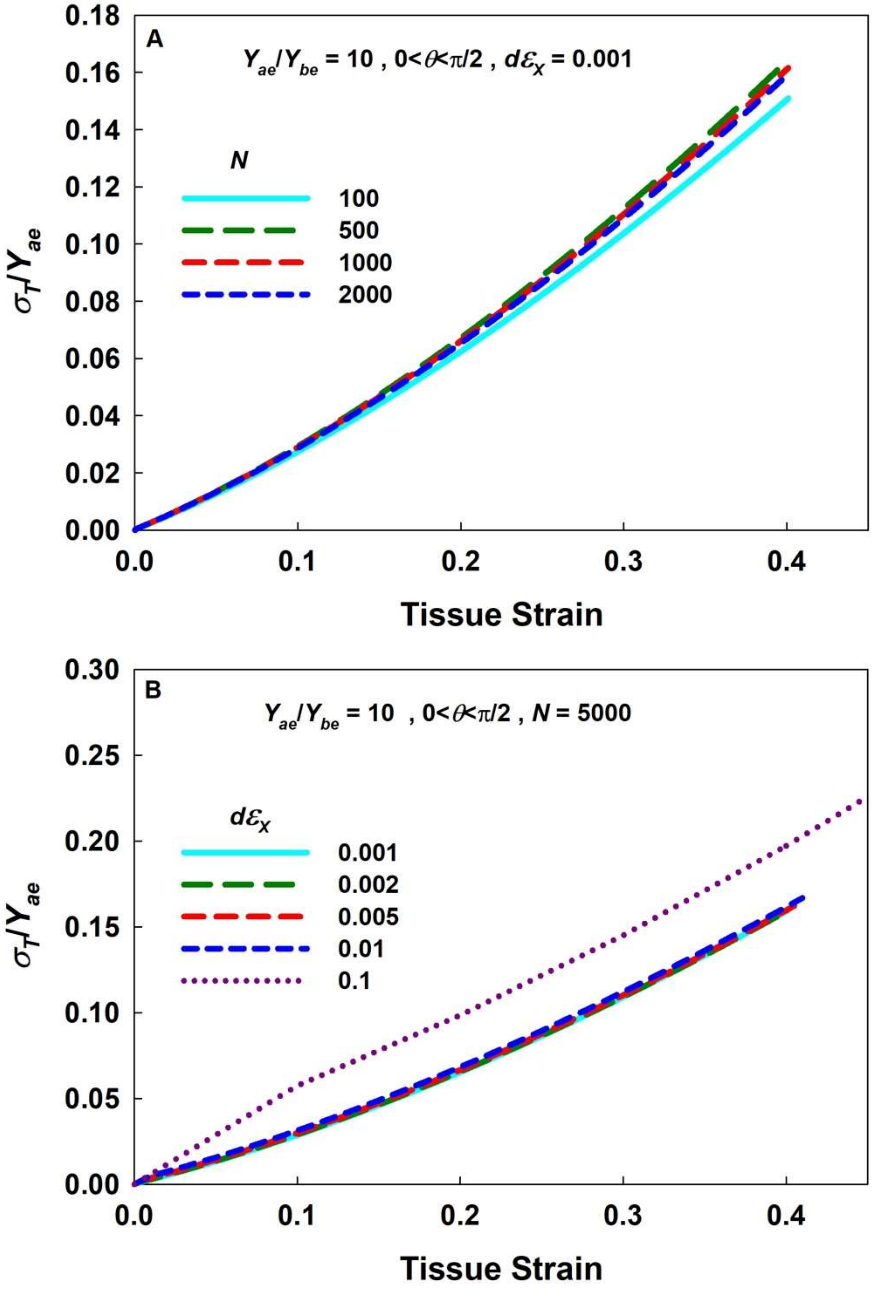

Figure 2: Effects of (A) alveolar wall number and (B) strain increment on tissue stress.
